# Fool’s gold, lost treasures, and the randomized clinical trial

**DOI:** 10.1186/1471-2407-13-193

**Published:** 2013-04-16

**Authors:** David J Stewart, Razelle Kurzrock

**Affiliations:** 1 Division of Medical Oncology, The University of Ottawa, Ottawa, Canada; 2 University of California San Diego Moores Cancer Center, San Diego, CA, USA

**Keywords:** Randomized clinical trials, Gold standard, Phase II trials, Drug combinations, Biomarkers

## Abstract

****Background**:**

Randomized controlled trials with a survival endpoint are the gold standard for clinical research, but have failed to achieve cures for most advanced malignancies. The high costs of randomized clinical trials slow progress (thereby causing avoidable loss of life) and increase health care costs.

****Discussion**:**

A malignancy may be caused by several different mutations. Therapies effective vs one mutation may be discarded due to lack of statistical significance across the entire population. Conversely, expensive large randomized trials may have sufficient statistical power to demonstrate benefit despite the therapy only working in subgroups. Non-cost-effective therapy is then applied to all patients (including subgroups it cannot help). Randomized trials comparing therapies with different mechanisms of action are misleading since they may conclude the therapies are “equivalent” despite benefitting different subpopulations, or may erroneously conclude that one therapy is superior simply because it targets a larger subpopulation. Furthermore, minor variances in patient selection may determine study outcome, a therapy may be discarded as ineffective despite substantial benefit in one subpopulation if harmful in another, randomized trials may more effectively detect therapies with minor benefit in most patients vs marked benefit in subpopulations, and randomized trials in unselected patients may erroneously conclude that “shot-gun” combinations are superior to single agents when sequential administration of personalized single agents might work better and spare patients treatment with drugs that cannot help them. We must identify predictive biomarkers early by comparing responding to progressing patients in phase I-II trials. Enriching randomized trials for biomarker-positive patients can markedly reduce required patient numbers and costs despite expensive screening for biomarker-positive patients. Available data support approval of new drugs without randomized trials if they yield single-agent sustained responses in patients refractory to standard therapies. Conversely, new approaches are needed to guide development of drug combinations since both standard phase II approaches and phase II-III randomized trials have a high risk of misleading.

**Summary:**

Traditional randomized clinical trials approaches are often inefficient, wasteful, and unreliable. New clinical research paradigms are needed. The primary outcome of clinical research should be “Who (if anyone) benefits?” rather than “Does the overall group benefit?”

## Background

### Unsustainable cost of our gold standard

Randomized controlled clinical trials (RCCTs) with survival endpoints are considered the gold standard of oncology research since death is an unambiguous endpoint, since longer survival is an important outcome, and since randomization is regarded as the optimal method to control for confounding variables and biases. However, it now costs $800M-$2B to bring a new drug from discovery to market, with gold-standard RCCTs being a major factor driving costs [1]. The average price is $47,000 per patient on phase III trials [2], with costs as high as $85,000 per patient in some studies [3], and with unwieldy research regulation driving much of the per-patient costs [4]. High research costs slow progress, since far fewer ideas can be tested with available resources, and delays in access to effective therapies can result in unnecessary loss of huge numbers of life-years [4]. Progress is further slowed by competition between large RCCTs for potentially available patients.

There are currently an estimated 800 new anticancer agents in clinical development [5], making it impossible to test most new drugs in more than a minority of situations where they might be useful [6]. While some of the 800 drugs in development have similar mechanisms of action, we cannot necessarily rely on testing with one member of a drug class to tell us what will happen with other members. For example, the BRAF inhibitor sorafenib is inactive against malignant melanoma with *BRAF V600E* mutations [7], while another BRAF inhibitor, vemurafenib, is highly active [8]. Consequently, requiring RCCTs for drug approval in each clinical situation means we are certain to miss numerous important new therapeutic opportunities at the same time that we are driving up health care costs. Current drug development paradigms are unacceptably wasteful and inefficient.

### The unfulfilled promise

The historical goal of RCCTs was step-wise incremental survival improvements that would initially convert incurability into occasional cures, followed ultimately by high cure rates, as happened with childhood leukemia [9]. RCCTs have contributed to improved adjuvant therapy and to modest prolongation of survival in the advanced disease setting for many malignancies. However, most cancers remain incurable when metastatic despite decades of successive minor incremental advances from RCCTs [10], and the impact of most new drugs has been small, with a median survival gain of only 2.19 months for drugs approved by the US FDA over the past 10 years [11]. The authors (neither of whom is a statistician) feel that faulty RCCT goals, endpoints, patient selection, and interpretation by clinicians, regulators and statisticians have played a role in slowing progress by facilitating and encouraging the pursuit of small advances, by prompting rejection of therapies that benefit subpopulations and by diverting resources away from other strategies [10].

### Fool’s gold

Early prospectors named ferrous sulfate “fool’s gold”. Its yellow color misled many into believing they had discovered great riches. We suggest that RCCTs are often fool’s gold- potentially deceptive and of limited value. Unquestionably, faulty conclusions can be drawn if one ignores the potential biases and errors that RCCTs are intended to prevent, but equally faulty conclusions can be drawn if the design and interpretation of RCCTs fails to adequately account for clinical and biological realities.

### Our goals

In this manuscript we will illustrate some of the ways in which RCCTs in unselected cancer patients may lead to erroneous conclusions. We will discuss why identification of predictive biomarkers early in the course of clinical drug development is very important, why use of response as the clinical endpoint is more efficient for biomarker discovery than is use of overall survival, and how early development of predictive biomarkers can speed drug development and markedly cut drug development costs. We will also discuss why traditional ways of doing phase II trials may no longer be appropriate, why drugs that lead to high response rates in defined populations should be approved without requirement for RCCTs, and why we need to change the way we assess drug combinations.

## Discussion

### Impact of molecularly distinct subgroups

Common cancers may be common since many mutations can cause them, and the probability of a particular therapy being beneficial may be strongly influenced by the presence of specific mutations [12]. Traditional RCCTs in unselected patients attempt to “overwhelm” molecular and clinical heterogeneity through randomization processes that are intended to achieve a balance between study arms with respect to factors that may impact outcome. However, this approach carries a substantial risk of generating erroneous conclusions unless most patients express the target of interest.

To illustrate this, we used GraphPad Prism 5 (GraphPad Software Inc, San Diego, CA) to perform *limited* simulations to generate examples of different ways in which erroneous conclusions can be drawn, with the nature of the error varying with the number of patients in the study, the size of a subpopulation with a target required for drug efficacy and the degree of benefit the drug conferred to patients with vs without the target [4]. We used the actual survival in 334 non-small lung cancer (NSCLC) patients as a “control” arm and a simulated group of 334 patients as the “experimental” arm. To provide a more accurate estimate of the probability of arriving at each type of erroneous conclusion with different sets of circumstances would have required several thousands of simulations, but that was not our objective. The probability of encountering each type of problem we address would differ if the simulations were run thousands of times using different data sets, but this would not alter the fact that there is a risk of each type of problem occurring if RCCTs are done in unselected patients, with the size of the risk varying inversely with the size of the subpopulation that might most benefit from the therapy.

### RCCTs may lead to loss of useful therapies

It is now widely recognized that effective therapies may be missed by RCCTs in unselected patients if the drug is only active in subpopulations. Various trial strategies have been proposed to address this issue [13-19]. To illustrate this problem, if we assumed that a required target was present in every 10^th^ patient (the approximate frequency of epidermal growth factor receptor [*EGFR*] mutations in Caucasians with NSCLC) and that therapy quintupled survival in those with target (in keeping with progression-free survival [PFS] gains when erlotinib is used as post-chemotherapy maintenance in *EGFR*-mutant NSCLC [20]), but was ineffective in those without target, the simulated “study” in unselected patients failed to achieve statistical significance (hazard ratio [HR]=0.85, p=0.16), and a new therapy tested in this way would not gain regulatory approval [4]. Since it costs on average $47,000 per patient on phase III trials [2], this study would cost $31,400,000, squander research resources, expose 90% of patients in the treatment arm to therapy incapable of helping them, and lead to potential loss of a “treasure” that is highly effective in subpopulations with target.

Despite the negative statistical outcome, investigators might conclude that the therapy was of value since survival curves diverged and 10% of patients responded. However, our past experiences tell us that many regulators, statisticians and clinicians would argue otherwise, and access to the drug would probably be at least substantially delayed, and there would be a high risk that the drug would be abandoned.

A case in point is gefitinib in NSCLC. Despite being of marked benefit in a subpopulation who experienced dramatic tumor regression, the survival gain was not statistically significant (p=0.09) in unselected patients [21], gefitinib was discarded for a period of time in North America and Europe, and the authors witnessed debates around why the related drug erlotinib was “effective” while RCCTs had “proven” gefitinib to be “ineffective”. While some investigators and statisticians would argue that this would be an incorrect conclusion, and would point out that the gefitinib [21] and erlotinib [22] survival curves are in fact extremely similar, one might well be concerned that a negative RCCT would introduce a strong bias against a drug, irrespective of issues with study design, and that this would hamper further study of the drug. A delay in approval of an agent with even modest activity can cause substantial loss of potential years of life [4]. While there is a growing appreciation of the risk of loss of valuable agents through RCCTs in unselected patients, these trials continue to be done.

### Large RCCTs may spawn low selectivity and poor cost-effectiveness

If we tripled patient numbers to 2,000 (current cost, $94,000,000 at $47,000 per patient) then survival gain and HR remained unchanged from the smaller simulated study, but increased statistical power yielded a p-value of 0.03 [4]. If the therapy only doubled survival in those with target, then more than 5300 unselected patients were required for significance (p=0.047, current cost $249,000,000) [4]. Since neither of these larger studies identified that only 10% of patients benefited, this expensive, potentially toxic therapy might well become the standard of care for the entire population, but would not help 90% of patients. With an α-error of 0.05, one study out of 20 of ineffective agents could be positive despite lack of any benefit.

The larger the RCCT, the smaller the benefit potentially detected and the poorer the cost-effectiveness. While this is an issue in oncology, it is an even bigger issue in other areas of medicine such as cardiology (we have referenced just a few of the very numerous examples) [23-26], where it is commonplace to detect statistically significant but extremely small absolute gains in survival by enrolling thousands of patients on studies, with a high proportion of studies being negative, despite the very large patient numbers enrolled.

Ocana et al. proposed that to reduce the risk of accepting therapies with only minimal benefit, a study should only be declared positive if the difference between the experimental arm and the control arm met a pre-specified size, in addition to the p-value being significant [27]. While this might reduce the risk of widely applying a therapy that only worked in a subpopulation, it would increase the risk of discarding a therapy that was of high value, but only in a subpopulation.

### Comparing therapies hitting different targets

**Figure 1:**
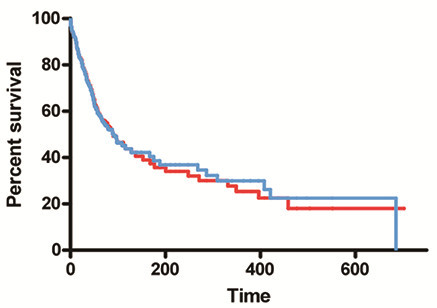
**Comparison of therapies hitting different simulated targets: Comparisons of a simulated therapy that quintupled survival in every 10**^**th**^** patient starting with patient number 10 to another that quintupled survival in every 10**^**th **^**patient starting with patient number 11 would erroneously conclude that the two therapies are equivalent (p=0.89), despite them being of benefit in completely different subpopulations.**

RCCTs are often designed to compare efficacy of two therapies. When we compared one simulated therapy that quintupled survival in every 10^th^ patient starting with patient number 10 to another that quintupled survival in every 10^th^ patient starting with patient 11, the statistical conclusion was that these therapies were equivalent (p=0.89) (Figure 1). However, this statistical conclusion is erroneous since the therapies are not equivalent: they are benefiting different subpopulations.

As a recent example, the NSCLC INTEREST study comparing gefitinib to docetaxel concluded that the two therapies were equivalent [28], but gefitinib gave a higher response rate and longer PFS than docetaxel in patients with *EGFR* mutations, while there was a trend towards docetaxel giving more responses and longer PFS in *EGFR*-wild-type patients [29]. It might have been reasonable to conduct a trial to assess the hypothesis that gefitinib would be the better drug in *EGFR*-mutant patients and that docetaxel would be the better drug in *EGFR*-wild-type patients, but it was not rational to conduct a study assessing whether the two drugs were equivalent. One could only conclude that they were equivalent by confining oneself to the statistical outcome and ignoring the fact that they work in substantially different ways.

Furthermore, if drug A hits a target present in 40% of patients while drug B hits a target present in only 20%, the statistical conclusion will be that drug A is the better drug [4,30]. Drug A is not better. It just hits a more common target. For the smaller subpopulation, drug B is the more effective therapy. If this goes unrecognized, then drugs that are important in smaller subpopulations will be discarded, the standard of care will be drug A, all patients will be treated with drug A despite it being incapable of helping the 60% who lack required target, and there will be no further advances if no target is more common than drug A target. It is illogical to use RRCTs in unselected patients to compare two agents hitting different targets.

### Minor variability in patient selection may sway outcomes

**Figure 2:**
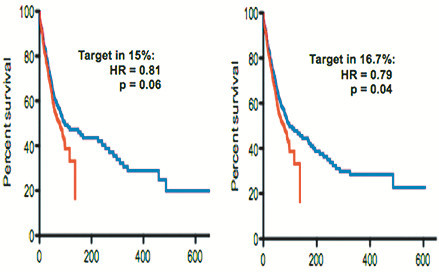
**Impact of minor changes in proportion of patients with target in simulated trials: If a new therapy quintupled survival in patients with a particular target, the 668-patient simulated study was negative if the target was present in 15% of patients (HR=0.81, p=0.06) but was positive if the target was present in just 11 more patients (16.7%) (HR=0.79, p=0.04).** Hence, very minor variations in study patient populations can determine whether a trial will be negative vs positive.

Small changes in patient characteristics may change study conclusions. If survival was quintupled in patients with a target present in 15% of patients, the therapy would be at risk of being discarded in our 668 patient simulated study since the study would not achieve statistical significance (HR=0.81, p=0.06), but would be accepted as effective if the target were present in just 11 more patients (16.7%) (HR=0.79, p=0.04) (Figure 2). For example, since both *EGFR* mutations [31] and *EML4*/*ALK* fusions [32] are more common in NSCLC non-smokers than in smokers, success of RCCTs of EGFR or EML4/ALK inhibitors in unselected patients could depend on minor variability in smoking incidence in the neighborhoods from which patients were recruited.

### Benefit in one subpopulation, harm in another

**Figure 3:**
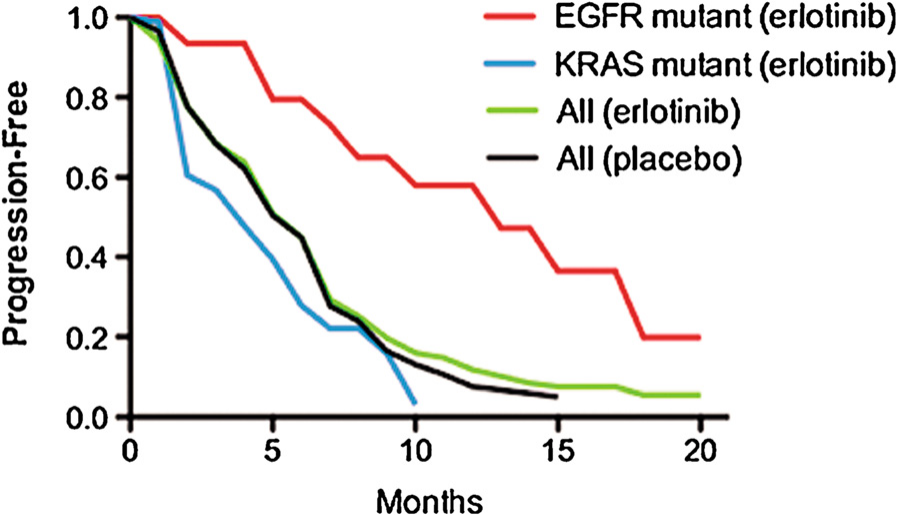
**Impact of benefit of erlotinib in one subpopulation vs harm in another: Despite substantial benefit in one subpopulation, a randomized trial may conclude that an agent is ineffective if it causes harm in a different subpopulation.** Erlotinib vs placebo were added to chemotherapy in NSCLC, [33] and the curves overlapped suggesting no impact of erlotinib (two center curves, redrawn from Herbst et al. [33]). However, on molecular assessment, erlotinib was associated with potential benefit in the 13% of patients with an *EGFR* mutation (p=0.09), but was associated with harm in the 21% of patients with *KRAS* mutations (p=0.03) (curves resynthesized using component parts from Eberhard et al. [34]).

RCCTs in unselected patients may also discard a therapy that is beneficial in one subpopulation if it is harmful in another. For example, NSCLC RCCTs adding erlotinib to chemotherapy concluded that erlotinib had little effect [33]. However, subsequent molecular assessments suggested that progression-free survival (PFS) (Figure 3) and response were increased by erlotinib in the 13% of patients with *EGFR* mutations but were significantly decreased by erlotinib in the 21% of patients with *KRAS* mutations [34]. Similarly, the anti-EGFR antibody cetuximab was associated with significant worsening of outcome when added to standard therapy in the treatment of *KRAS*-mutant metastatic colorectal cancer [35], while it may improve outcome in *KRAS*-wild-type patients [36].

### Types of gains detected by RCCTs

**Figure 4:**
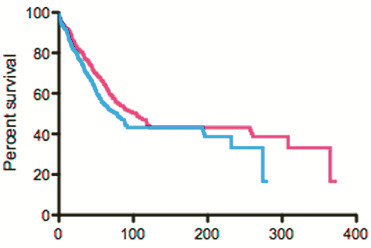
**Therapy giving minor benefit in all patients achieved significance in simulated trial: In a 668 patient simulated study, a therapy that increased survival by 33% in all patients was judged to be effective (HR=0.80, p=0.03) (survival curves presented here), while a therapy that quintupled survival in 10% of the patients was judged ineffective (HR=0.85, p=0.16, see Figure **1** from Stewart, Whitney and Kurzrock **[4]**).**

RCCTs in unselected patients may be less effective at detecting large gains in subpopulations than at detecting small gains in the overall population [4]. As noted above, our 668-patient simulated trial of a drug that quintupled survival in 10% of patients (e.g., increasing median survival from 2 months to 10 months) would fail to achieve statistical significance, while a simulated trial of therapy that increased survival in all patients by 33% (e.g., from a median of 2 to 2.7 months, a gain of 21 days, similar to the statistically significant but clinically minute 11-day median survival gain seen when erlotinib was added to chemotherapy in the treatment of metastatic pancreatic cancer [37]) did achieve significance (HR=0.80, p=0.03, Figure 4). Despite these different statistical conclusions, the life-years gained across a total population of 100 patients might be higher with the quintupling of survival in a 10% subpopulation than with an increase in survival of 33% in each member (6.7 vs 5.8 life-years in our simulated examples). Overall, conclusions reached by RCCTs in unselected patients may be appropriate if the therapy hits a target present in most patients, but will be problematic for drugs hitting less common targets.

### False negatives and positives due to unrelated factors

While survival has the advantage that it is a very precise endpoint, it has the disadvantage that unrelated factors may impact it to a greater extent than they impact response or PFS, and a therapy may fail to be associated with a survival advantage for reasons unrelated to therapy efficacy [38]. Specifically, the probability of detecting a significant survival benefit can be blunted by the impact of major comorbidities, cross-over to the study agent, long post-progression survival for any reason [39], or palliative care (which can prolong survival [40]). Conversely, some therapies may correlate with survival for reasons that have nothing to do with their anticancer effects. For example, adjuvant BCG prolonged survival of colorectal cancer patients by reducing deaths from heart disease without having any apparent impact on the patients’ cancers [41]. In any trial with a survival endpoint, detailed information should be collected following discontinuation of study therapy to help better assess the impact of subsequent therapy and of unrelated events.

### Randomized discontinuation designs

It has been suggested that for cytostatic agents, assessing further time to progression after randomizing stable patients to continue vs stop a therapy could provide proof of benefit. For example, this approach demonstrated potential benefit of sorafenib in metastatic renal cell carcinoma [42]. However, waterfall plots from this study suggest that approximately 70% of treated patients had at least some degree of tumor shrinkage, and it is debatable whether the addition of a randomized discontinuation approach added much value. This approach also requires relatively large numbers of patients, and it has been questioned whether it is ethical to withdraw a therapy that is controlling a patient’s cancer [43]. Furthermore, while this approach is intended to assess the benefit of stable disease, stable disease (unlike response) does not correlate with PFS or survival for either targeted agents or chemotherapy [11,44], and we agree [10] with Fojo and Noonan [11] that aiming for stable disease is aiming too low.

### Chemotherapy

Publications have stressed the importance of new clinical trial designs for targeted agents [13-19]. However, new trial designs might be as important for chemotherapy. For most adult malignancies, only a subpopulation of patients responds to most chemotherapy single agents. Differential sensitivity is potentially due to discoverable molecular differences, and there are many factors that influence tumor sensitivity [45,46]. Defining the factors that are important clinically could convert chemotherapy into targeted therapy.

### RCCTs to discover predictive biomarkers

A variety of strategies have been described to discover or validate predictive biomarkers [47-50]. In some RCCTs, post-hoc analysis (using survival as the clinical endpoint) has been done to identify biomarkers predicting drug benefit [51,52], and we have heard it argued that their use in discovery of important biomarkers is one reason why RCCTs are of value. However, while RCCTs may be used in a variety of ways to validate biomarkers [50], RCCT post-hoc analyses have been at best only modestly successful as a strategy to discover clinically important biomarkers that can permit rational patient selection.

Various adaptive designs have also been proposed. For example, the probability of a patient with a given biomarker being randomized to receive an agent may increase if earlier marker-positive patients benefited from the agent [13]. The major issue with this approach is that only a relatively small number of biomarkers can be assessed. While adaptive designs may be useful in validating predictive biomarkers [50], they have not yet proven to be an efficient way of discovering previously unappreciated biomarkers. An adaptive signature approach, wherein outcomes with a therapy vs control group are compared in different biomarker groups [16], may possibly prove more useful, although this remains to be determined.

### Identification of markers correlating with tumor regression in phase I-II trials

There are several potential advantages to using durable tumor regression in phase I, II and III trials (and not survival) as the outcome variable in discovering predictive biomarkers [4,50]. Since tumors do not usually shrink spontaneously, tumor shrinkage generally indicates drug effect, one can tell which individual patients benefitted, and you only require a few weeks or months of patient follow-up time to determine response. As noted above, survival has the advantage of being a more precise endpoint than response, but it has the distinct disadvantages of being impacted by a variety of factors unrelated to therapy efficacy, one cannot tell which patients actually benefited from therapy, and it requires several months or years of patient follow-up time. Generally, far larger patient numbers are needed to detect an association of a biomarker with survival than with response. For example, benefits of cetuximab and panitumumab in colorectal cancer and benefits of the EGFR tyrosine kinase inhibitors (TKIs) erlotinib and gefitinib in NSCLC are respectively associated with presence vs absence of *KRAS* and *EGFR* mutations. Across a range of colorectal cancer and NSCLC studies, p-value for association of response with mutation status was usually more significant than association of overall survival with mutation status (Table 1) [35,36,52-70], in keeping with increased statistical power with a response endpoint. PFS also generally did better than overall survival, and was almost as good as response (Table 1).

**Table 1:** Differences in p values when using response vs PFS vs overall survival to assess association of outcomes with biomarkers

**Agent**	**No. patients**	**P values**^**a**^		
		**Response**	**PFS**	**Survival**
P values for differences in outcome for *KRAS* wild type vs *KRAS* mutant colorectal cancer patients treated with single agent monoclonal antibody:				
Panitumumab [52]	427	<0.0001^b^	<0.0001	>0.05
Cetuximab [53]	30	0.0003	NR^c^	0.016
Cetuximab [54]	108	0.000001	0.074	0.020
P values for differences in outcome for *KRAS* wild type vs *KRAS* mutant colorectal cancer patients treated with combined monoclonal antibody and chemotherapy:				
Cetuximab [55]	110	0.0024	0.0009	NR
Cetuximab [56]	69	0.021	0.021	0.15
Cetuximab [35]	256	0.03	0.04	0.06
Cetuximab [57]	540	0.03	0.07	0.44
Cetuximab [58]	88	0.024	0.003	0.0004
Cetuximab [59]	48	0.144	0.048	NR
Cetuximab [36]	315	<0.001	<0.001	0.12
Cetuximab [60]	67	0.07	0.14	0.047
Cetuximab [61]	1,063	0.0005	0.0028	0.0463
Cetuximab [62]	58	0.027	0.024	0.107
P values for differences in outcome for *EGFR* wild type vs *EGFR* mutant NSCLC patients treated with single agent tyrosine kinase inhibitor:				
Gefitinib or erlotinib [63]	223	<0.0001^b^	<0.0001	0.002
Gefitinib [64]	57	0.002	NR	0.11
Gefitinib [65]	100	0.0017	NR	0.0135
Gefitinib [66]	68	0.0001	NR	0.001
Gefitinib [67]	66	<0.0001	<0.0001	0.0001
Gefitinib [68]	83	0.001	0.002	0.02
Erlotinib [69]	36	0.006	NR	0.045
Erlotinib [70]	116	0.035	NR	0.47

a. Comparison of outcome in mutant vs wild type or p value for interaction between treatment arm on a comparative study and mutation status.

b. Calculated from published data using Fisher’s exact test.

c. NR = not reported.

Furthermore, since survival is impacted by both predictive factors (linked to therapy efficacy) and prognostic factors (linked to tumor aggressiveness, irrespective of therapy), RCCTs comparing patients with vs without a factor in a therapy arm vs a control arm are needed to differentiate predictive from prognostic factors if using a survival endpoint [71], and this further increases the number of patients required to discover or validate a predictive biomarker. Response is likely to be much less influenced by prognostic factors than is survival, and hence does not require RCCTs to differentiate predictive factors from prognostic factors.

For some agents, assessment of tumors from patients with responses in phase I or II trials led to the discovery of important, previously-unappreciated biomarkers (e.g., *EGFR* activating mutations for erlotinib and gefitinib in NSCLC [72,73], *EML4*/*ALK* fusions for crizotinib in NSCLC [74], and *KRAS* mutation status for cetuximab in colorectal cancer [53]). Other response observations have suggested potentially important biomarkers that are currently being assessed further (e.g., *DDR2* mutations [75] and inactivating *BRAF* mutations [76] for dasatinib in NSCLC). Phase I and II trials with relatively small numbers of patients have also supported the importance of other biomarkers that were a priori hypothesized to be important (e.g., estrogen receptors for tamoxifen in breast cancer [77], Her-2/neu overexpression for trastuzumab in breast cancer [78], *BCR*/*ABL* fusion genes for imatinib in chronic myelogenous leukemia [79], *c*-*KIT* mutations for imatinib in gastrointestinal stromal tumors [80], *BRAF v600E* mutations for selected BRAF inhibitors in malignant melanoma [81], and PD-L1 expression for an anti-PD-1 antibody [82]). Furthermore, patient outcomes were substantially better in phase I trials where patients were selected based on putative biomarkers [83].

Currently available data for selected biomarkers suggest that a high proportion of biomarker-positive patients may respond, and a high proportion of patients who do not achieve a RECIST response may nevertheless have measurable tumor regression. We manually measured available waterfall plots to estimate the proportions of patients with tumor shrinkage of >30%, >10% and >0% for erlotinib and gefitinib in *EGFR*-mutant NSCLC patients [63,84-89], for crizotinib in NSCLC patients with *EML4*/*ALK* fusion genes [90], and for vemurafenib in malignant melanoma patients with *BRAF V600E* mutations [8]. We found that 50-84% of biomarker-positive patients had >30% reduction in tumor diameter, 80-95% had >10% reduction, and 90-100% had at least some degree of measurable reduction in tumor size (Table 2). Conversely, on placebo or best supportive care (BSC) arms of other trials (Table 3), RECIST objective responses were uncommon (median, 0%, range, 0-4%) [21,22,52,91-112], and the proportion of patients with measured reduction in tumor diameter of >10% was low (median, 6.6% of patients, range 1-9%, as estimated from manual measurements of available waterfall plots) [52,98-100,103,107,112]. The low proportion of patients who were judged to have tumor regression of >10% with placebo or BSC is in keeping with the observation that on repeat scans done 15 minutes apart in 30 patients with lung lesions, there was a decrease in size of >10% in only 7.8% of patients with repeat measurement, and no patient had a decrease of greater than 25% [113].

**Table 2:** **Proportion of patients with reduction in tumor size** >**0**%, >**10**% **and** >**30**%, **for patients with vs without selected resistance**/**sensitivity biomarkers**

**Drug**	**Tumor type**	**Biomarker**	**% Biomarker-positive patients with tumor shrinkage**^**a**^			**% Biomarker-negative patients with tumor shrinkage**^**a**^		
			**Tumor shrank > 0%**	**Tumor shrank > 10%**	**Tumor shrank > 30%**	**Tumor shrank > 0%**	**Tumor shrank > 10%**	**Tumor shrank > 30%**
Panitumumab [52]	Colorectal	*KRAS* wild type	57%	50%	25%	4%	1%	0%
Erlotinib [84]	NSCLC	*EGFR* mutant	100%	83%	72%	28%	12%	8%
Erlotinib [85]	NSCLC	*EGFR* mutant	100%	83%	50%	31%	17%	3%
Erlotinib [86]	NSCLC	*EGFR* mutant	90%	88%	76%			
Erlotinib [87]	NSCLC	*EGFR* mutant	100%	80%	70%			
Gefitinib [88]	NSCLC	*EGFR* mutant	95%	95%	63%	72%	38%	10%
Gefitinib [89]	NSCLC	*EGFR* mutant	91%	82%	55%	67%	33%	0%
Erlotinib or gefitinib [63]	NSCLC	*EGFR* mutant	97%	91%	84%	45%	22%	9%
Crizotinib [90]	NSCLC	*EML4*/*ALK* fusion	94%	88%	69%			
Vemurafenib [8]	Melanoma	*BRAF* V600E	96%	93%	76%			

a. Calculated from manual measurement of available waterfall plots.

**Table 3:** **Response rates and proportion of patients with measured tumor shrinkage** >**10**% **in single agent placebo or best supportive care arms of randomized trials**

**Tumor type**	**RECIST response %**	**% of patients with measurable tumor shrinkage >10%**^**a**^
NSCLC [21]	1	NA^b^
NSCLC [22]	<1	NA
NSCLC [91]	0.7	NA
NSCLC [92]	1	NA
Colorectal [52]	0	1
Colorectal [93]	0	NA
Colorectal [94]	0	NA
Renal cell [95]	2	NA
Renal cell [96]	0	NA
Renal cell [97]	3	NA
Renal cell [98]	0	4
Renal cell [99]	0	5.5
Hepatocellular [100]	0	9
Hepatocellular [101]	3	NA
Hepatocellular [102]	1.3	NA
Head and neck cancer [103]	0	7
Head and neck cancer [104]	1	NA
Head and neck cancer [105]	3	NA
Transitional cell [106]	0	NA
Pancreatic neuroendocrine [107]	0	7
Pancreatic neuroendocrine [112]	2	6.6
Prostate [108]	4	NA
Sarcoma [109]	0	NA
Medullary thyroid [110]	1	NA
Mesothelioma [111]	1.7	NA

a. Calculated by manual measurement of published waterfall plots.

b. NA = not available.

Hence, where it would be helpful to increase statistical power, it may be appropriate to use proportion of patients with >10% tumor regression to compare biomarker-positive to biomarker-negative patients when assessing the biomarker as a potential predictive factor. In addition, this type of approach might help estimate the proportion of patients who have an important undiscovered predictive biomarker. For example, in *EGFR*-wild type NSCLCs, 0-10% of patients (median, 8%) experience a >30% reduction in tumor diameter with erlotinib or gefitinib, and 12-38% (median, 22%) experience a >10% reduction in tumor diameter (Table 2) [63,84,85,88,89]. If we assume based on the above observations that tumor regression >30% usually (but not always) indicates drug efficacy rather than measurement error, that most patients with an important biomarker who do not achieve partial remission will nevertheless have some degree of tumor shrinkage, and that approximately 5-10% of the time a measured tumor regression of >10% will be due to measurement error rather than being due to drug benefit, then we might estimate that approximately 10-15% of *EGFR*-wild type NSCLCs have a currently undefined sensitizing target that could help explain apparent benefit of EGFR TKIs in patients from groups that ordinarily do not respond to these agents [114].

Conversely, of patients treated with panitumumab for *KRAS* wild-type colorectal cancer, 17% achieved partial remissions by RECIST criteria [52], 25% had tumor regression of >30% (estimated from measurement of waterfall plots) and 50% had tumor regression >10%. Less than 1% of *KRAS*-mutant tumors shrank by >10% (Table 2) [52]. This suggests that the still-unrecognized “true target” for panitumumab (and cetuximab) is present in 30-40% of *KRAS* wild-type colorectal cancers, and in almost no *KRAS*-mutant colorectal cancers. It appears that this hypothetical “true target” is also generally absent in tumors with *BRAF* mutations [115] or *PIK3CA* mutations [116]. Overall, we have been less successful at finding targets associated with a high probability of benefit from monoclonal antibodies than with some small molecules. We suspect that this is primarily because there have been insufficient molecular assessments comparing patients with vs without tumor regression on single agent therapy with monoclonal antibodies, although it remains possible that there are biological reasons instead.

### Cytostatic agents

It has been argued that a response endpoint would not be informative with cytostatic agents since cytostatic agents might confer benefit without inducing tumor shrinkage [42]. However, a high proportion of targeted agents that were initially anticipated to be cytostatic can induce tumor shrinkage, including antiangiogenic agents such as bevacizumab [117-122]. Hence, tumor shrinkage could also potentially be a valid endpoint for biomarker identification for purportedly cytostatic agents. On the other hand, response may be somewhat less reliable with immunotherapeutic approaches since in some instances, there may be delayed tumor shrinkage, with or without a period of continued tumor growth prior to onset of sustained tumor shrinkage [82,123], or there may be prolongation of survival without response or improvement in PFS [124].

### Continuously variable and graded biomarkers vs dichotomous biomarkers

In searching for useful biomarkers, dichotomous (present vs absent) factors (e.g., gene mutation, amplification, deletion or expression) may be easier to use than continuously variable or graded markers (e.g., degree of gene or protein expression). Continuously variable markers may be challenging due to measurement variability, time-dependent expression fluctuations and biologically irrational use of cut-points to dichotomize patients into low vs high benefit groups, thereby classifying 51^st^ percentile patients as different from 49^th^ percentile patients but equivalent to 99^th^ percentile patients. There are few examples where continuous variables have proven helpful clinically in predicting benefit in individual patients unless the cut point is placed at the extreme of almost no expression vs any expression. For example, breast cancers with just 1-10% of cells that are positive for estrogen receptors respond far better to tamoxifen than do estrogen-receptor-negative cancers and respond almost as well as highly positive cancers [125]. Conversely, very high EGFR expression by immunohistochemistry (IHC) may predict NSCLC benefit from cetuximab [126], although this requires further confirmation. While very high Her-2/neu expression by IHC appeared to predict trastuzumab benefit in some studies [78,127,128], other authors have concluded that IHC is not as reliable as FISH assessment of gene amplification (any vs none) in predicting efficacy [129].

We would anticipate that continuous variables would be most likely to be useful if there is a nonlinear relationship between expression and benefit (as noted above for estrogen receptors), such that a true benefit threshold can be identified. If the relationship between benefit and marker expression is linear, then using cut points could successfully validate that the marker was significantly associated with outcome, but it would be less useful as a guideline for making therapeutic choices. With linear relationships, instead of using cut points, we should consider models that enable estimation of a predicted patient-specific probability or degree of benefit, analogous to the approach used by Oncotype Dx to assign a specific prognostic score and probability of benefit from adjuvant chemotherapy to patients with resected breast cancer [130].

### For biomarkers, is repeat biopsy required?

While doing repeat biopsies to assess biomarkers is feasible in patients with metastatic disease [131], the benefit derived may vary with the patient’s situation. Contrary to the findings of Bai et al. that tumor *EGFR* mutation status changed after chemotherapy [132], the experience with several biomarkers to date (including with *EGFR* mutation status) suggests that formalin-fixed paraffin-embedded (FFPE) archival tissues from initial diagnostic biopsies may be a very adequate source of tissue for biomarker assessment, even if the patient has undergone chemotherapy between tissue acquisition and administration of the targeted therapy [53,72-74,77,78,80,81].

On the other hand, if the patient has undergone targeted therapy and has responded to it, the characteristics of the residual cancer at the time of progression may be substantially different from the earlier diagnostic biopsy since the targeted therapy may have suppressed sensitive clones that made up the bulk of the tumor at diagnosis, and may have permitted outgrowth of resistant sub clones that may have made up an undetectably small proportion of the cells in the original tumor biopsy. An example is the selection of resistant *T790M*-positive cells by treatment of *EGFR*-mutant NSCLC with EGFR TKIs [133]. Hence, for assessment of tumor characteristics in residual or recurrent cancers after initially successful treatment with targeted agents, early experience suggests that fresh biopsies may be required.

In addition, while archival FFPE tissues may be quite suitable for assessment of gene mutations and amplification, tissue processing and fixing methods may reduce accuracy of IHC assessments for at least some biomarkers [129], raising the possibility that carefully processed repeat biopsies may be required if therapy is being directed by IHC assessments.

In obtaining fresh biopsies, core biopsies show tissue architecture but fine needle aspirates (FNAs) have the advantage that specimens consist mainly of tumor cells with little stroma since tumor cells are less cohesive and more easily aspirated than stromal cells [134]; hence, laser-capture microdissection is unnecessary with FNAs to obtain a relatively pure tumor cell population, and FNAs work well for tumor molecular assessment provided fixation is with formalin (yielding FFPE cell blocks) rather than with alcohol. FNAs are easier to obtain than core biopsies for tumors in some locations (e.g., with ultrasound-guided endobronchial sampling of mediastinal lymph nodes).

### Patient selection using biomarkers correlating with response can reduce costs

The extent to which selecting patients based on a biomarker may improve efficiency of phase III trials will depend on the extent to which the treatment benefits different patient subpopulations, the proportion of the population that belong to sensitive subpopulations and the reliability of the assay [135]. If the subpopulation possessing a target that is required for benefit is relatively small and if the therapy is of limited benefit in those without the target, then the number of patients required for a phase III trial to demonstrate benefit of a new therapy may be much smaller if one selects for the target than if one uses unselected patients [50]. In keeping with this, in our simulations, if we had first identified the important target based on differences between a few responders and non-responders in phase I-II trials and if we then confined RCCTs to patients with this target, only 16 patients would have been needed in a phase III trial with a survival endpoint to confirm drug efficacy in the extreme example of drug quintupling survival in those with target (HR=0.2, p<0.02), while 84 patients would be needed if the drug only doubled survival in those with target (HR=0.5, p<0.04) [4].

If it cost $10,000 per patient to screen 160 patients to find the 16 needed where drug quintuples survival, then the trial (including screening) would cost $2,350,000, while it would cost $12,348,000 to screen 840 patients and study 84 in the situation where drug doubled survival. In both cases, RCCT patient numbers would be reduced by >98% and trial costs by >94% compared to those required to detect benefit using unselected patients. Post-marketing drug utilization would be reduced by 90%. Hence, even large upfront investments in defining targets linked to response in early trials could prove highly cost-effective. It is stressed that while these examples are illustrative only, and that while actual reduction in costs and resource utilization in individual situations might be much less than suggested by our examples, they may nevertheless be substantial.

### Design of phase II trials

By the Simon 2-stage design [136] and similar approaches, 14–15 patients are typically entered on the first stage of a phase II trial. If responses are seen, then the phase II trial is expanded, while if no responses are seen, the study is stopped since there will then be less than a 5% probability that the true response rate will be 20% or higher. While RCCTs in unselected patients are problematic in the era of targeted therapies, the Simon 2-stage phase II trial design may also no longer be appropriate in many situations. Since uncommon tumors may be uncommon as a consequence of having only a small number of potential driving mutations [12], and since a high proportion of patients with a relevant mutation may respond to a targeted agent (as noted above), then relatively small phase II trials may be reasonable with uncommon malignancies.

However, this approach is probably not appropriate for common malignancies, where there may be a large number of different driving mutations, and entering 14–15 unselected patients with common malignancies on a phase II trial should not be used as a basis for rejecting the agent. The options need to include either much larger phase II trials, with adjusted early stopping rules and with tissue acquisition on all patients to enable molecular characterization of any responding patients, or else the phase II trials need to be limited to patients who have already been characterized, with enrichment for potential targets. However, the factor(s) that we think will be important for drug activity based on preclinical models may end up not being the ideal or relevant target, as was the case with EGFR IHC expression for EGFR TKIs in NSCLC and for cetuximab in colorectal cancer [137]. Furthermore, there may be very important targets that are not identified by preclinical data, such as *EML4*/*ALK* fusions in crizotinib-treated NSCLC [74], and a drug may be effective against more than one target. For example, in addition to being effective against *EML4*/*ALK* fusions, crizotinib may also be effective against NSCLCs with *ROS1* fusion genes and with *c*-*Met* amplification [138,139]. Hence, our phase II catchment strategies have to be sufficiently broad to compensate for the fact that we may potentially get it wrong, although the preclinical data did correctly predict activity in several instances [77-82].

Since presence of a target may be associated with a very high probability of tumor regression, one option would be to undertake large phase II trials (with tissue acquisition), but to stop accrual of patients with specific mutations if none of the first 3–4 patients had tumor regression >10%, while accrual would be continued for other mutations. It would be important to consider the actual type of mutation (and not just which gene was mutated), since in a particular gene, one type of mutation may not be equivalent to another type. For example, only specific *EGFR* mutations sensitize cells to EGFR TKIs [140], different *p53* mutations have markedly different effects on drug efficacy [141], and different *KRAS* mutations drive activation of different downstream pathways [142].

### Should drugs be approved based on phase II response?

As noted, survival is our gold standard outcome. Since there are numerous examples of response not translating into a survival advantage, many investigators regard response as a suboptimal surrogate outcome. On the other hand, above we outlined problems with RCCTs with a survival endpoint in unselected patients, response rate in single-agent phase II trials is a highly reliable predictor of eventual regulatory approval (p=0.005) [143], response correlates very strongly with survival for both chemotherapy [44,144] and targeted agents (p<0.0001) [44], and the overwhelming majority of anticancer agents approved by the FDA based on sustained responses (without RCCTs) have withstood the test of time [145]. The available data indicate that if a single agent can induce a reasonable rate of sustained response in pretreated patients from a defined population then it should be approved for use, without the requirement for RCCTs.

The probability of achieving a response with a placebo-equivalent ineffective agent is very low (Table 3). The larger the lesion that one measures, the more precise the measurement, and precision of measurement of even small lesions can be improved by requiring measurement of multiple (e.g., 4 or more) target lesions in a given patient [113]. By reducing drug development costs and by speeding access to effective new therapies, approval of agents based on single-agent phase II data could reduce the costs of marketed agents, greatly increase the number of agents that can be tested using available resources, and improve patient outcomes [4].

### Approach to combining new agents with standard therapies

Chemotherapy combinations are the standard of care as first-line treatment for most malignancies. While the data support single-agent response rates in pretreated patients as a basis for drug approval, combinations are problematic [146,147]. Outcome with a standard regimen may be quite variable. Hence, if one sees a better-than-anticipated outcome when one adds a new agent to a standard regimen, one cannot tell whether this is due to variability in efficacy of the base regimen vs being due to benefit from the new agent. To address this issue, RCCT phase II trials have been proposed as a tool to help select combinations worthy of further assessment [146,147], but in addition to the problems already noted above, RCCTs risk overstating the value of combinations. For example, if drug A hits a target present in 25% of patients and drug B hits a different target present in 20%, then a RCCT comparing A+B to A alone might suggest that the combination is superior, with benefit in 45% of patients. A+B then becomes the new standard of care. However, with A+B, all patients would potentially be receiving one expensive, toxic drug that was not helping them (since patients with target A would not benefit from drug B, and patients with target B would not benefit from drug A), and the 55% of patients with neither target A nor target B would not benefit from either agent. A better strategy would be to define who will benefit from A, who will benefit from B, and to only treat patients with the drug(s) that would be expected to work in them, rather than using a shotgun combination approach.

Furthermore, if the patient had both targets A and B, the preferred option might be sequential therapy, since giving single agents might be less toxic than combinations, since higher doses of each drug might then be feasible, and since higher doses may give enhanced efficacy for some single agents [148]. With this sequential approach, response rates and PFS on each individual single agent might be lower than with A+B combined, but there might not be any detrimental impact on survival, provided patients did receive the agent(s) for which they had targets. As an example, combination chemotherapy in breast cancer increased response rates compared to sequential single agents, but had no beneficial impact on survival, and survival may even have been longer with sequential therapy for some subgroups [149].

Potential synergism is one rationale for combinations, but there are few clinical examples of true drug synergism. There is also a rationale for combining targeted agents that hit complementary pathways, but to date there are few confirmed examples of combined targeted agents being better than single agents, and toxicity is often increased. Combinations might best be assessed based on presence of demonstrable preclinical synergism across a wide range of cell lines, or based on presence of known targets for both agents, or based on a potential for the second agent being able to block an alternate growth pathway or resistance mechanism that might protect against the first agent. It is important to have a biologically-based hypothesis with respect to what one expects to see with the combination, and that the trial design is appropriate to test the hypothesis.

**Figure 5:**
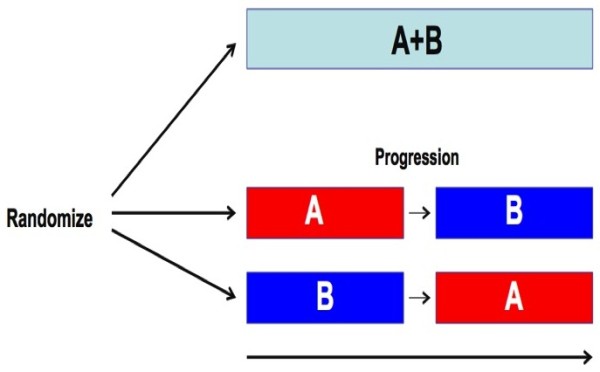
**Suggested trial structure for assessment of combinations: Combination A+B would be compared to A followed at progression by B and to B followed at progression by A.** Endpoints might include: 1) Time to progression on A+B vs time to progression from the initiation of the first single agent until completion of the second single agent; 2) A+B would be compared to each of A alone or B alone with respect to time to progression and maximum response; 3) Overall survival on the 3 arms; 4) Ability of B to suppress emergence of specific resistant clones while on A. 5) Of very high importance would be the development of molecular signatures that predict unique benefit of A alone, B alone, A+B combined, and the A→B/B→A sequences.

To assess combinations, we would suggest the following approach: If one hypothesizes that drug B will be synergistic with drug A, then randomize patients to A followed at progression by B (the “A → B” arm) vs B followed at progression by A (since there can be schedule-dependent synergism vs antagonism) [150] (the “B → A” arm) vs A combined with B (the “A+B” arm). For potential study endpoints see Figure 5. This would allow assessment of whether A+B is better than either single agent alone, and whether it is better than sequential therapy. It would also be important to have baseline tissue to permit molecular assessment of which patient subpopulations (if any) actually benefit from combination therapy.

In addition to improved PFS and survival, one would also expect to see increased tumor regression with effective combinations. Even purportedly cytostatic agents such as bevacizumab can significantly increase tumor response rates when added to chemotherapy (Table 4) [151-155]. For new anticancer drugs approved by the FDA between 2002 and 2012 [11], all except one that significantly improved survival when added to standard therapy also significantly improved response rates (Table 4) [37,151-171].

**Table 4:** **Gain in response rate vs gain in survival for anticancer drugs approved by FDA 2002**-**2012**

**Drug**	**Tumor type**	**Gain in response rate**	**p**	**Gain in OS, months**	**p**
Oxaliplatin [156,171]	Colorectal	48% vs 32%	0.006	5.6	<0.001
Pemetrexed [157]	Mesothelioma	41.3% vs 16.7%	<0.0001	2.8	0.02
Bevacizumab [152]	Colorectal	44.8% vs 34.8%	0.004	4.7	<0.0001
Gemcitabine [158]	Breast	41.4% vs 26.2%	0.0002	2.8	0.0489
Erlotinib [37]	Pancreas	8.6% vs 8%	NS^a^	0.33	0.038
Docetaxel [159]	Gastric	37% vs 25%	0.01	1.9	<0.001
Topotecan [160]	Cervix	27% vs 13%	0.004	2.9	0.017
Bevacizumab [153]	Colorectal	22.7% vs 8.6%	<0.0001	2.1	0.0011
Gemcitabine [161]	Ovary	42.7% vs 30.9%	0.0016	0.7	0.83^b^
Bevacizumab [151]	NSCLC	35% vs 15%	<0.001	2	0.003
Docetaxel [162]	Head and Neck	68% vs 54%	0.006	3.3	0.02
Lapatinib [163]	Breast	22% vs 14%	0.09	NA^c^	0.72 ^b^
Lapatinib [164]	Breast	23.7% vs 13.9%	0.017	0.3	0.18 ^b^
Temsirolimus [165]	Renal	8.1% vs 4.8%	NS	1.1	0.70 ^b^
Ixabepilone [166,167]	Breast	35% vs 14%	<0.0001	1.8	0.19 ^b^
Bevacizumab [154,155]	Renal	31% vs 13%	0.0001	2.0	0.33 ^b^
Lapatinib [168]	Breast (Her-2/neu +ve)	28% vs 15%	0.021	1.0	0.11 ^b^
Trastuzumab [169]	Gastroesophageal	47% vs 35%	0.0017	2.7	0.0046
Cetuximab [170]	Head and neck	36% vs 20%	<0.001	2.7	0.04

a. NS: not significant.

b. PFS differed significantly.

c. NA: not available.

If one were anticipating additivity of A and B, it would be important to use relevant biomarkers to select patients with target for both agents. On the other hand, if one were hypothesizing synergism (e.g., through concurrent blocking of alternative signaling pathways), selection based on anticipated sensitivity to single-agents might be less important. For example, cisplatin radiosensitization may be even greater in cisplatin-resistant cell lines than in cisplatin-sensitive cell lines [172]. One might also require few patients if there is synergism, since the combination should give much better activity than either “A → B” or “B → A” if synergism is actually present.

If one hypothesizes that B will prevent emergence of a resistant sub clone X during treatment with A (e.g., prevention of emergence of a *T790M*-mutant clone during treatment with an EGFR inhibitor [133]), then one might use the “A → B” vs “B → A” vs “A+B” approach, but with tumor biopsy at a set time point after starting A in each of the 3 arms to assess ability of B to prevent emergence of sub clone X, as well as to identify other sub clones that might preferentially appear in the presence of B. For example, while emergence of *T790M*-mutant sub clones are one potential mechanism of acquired resistance of *EGFR*-mutant NSCLC to EGFR TKIs, emergence of resistant sub clones with *c*-*Met* amplification may also occur [173].

Where one hypothesizes that B will be effective against a clone X that is resistant to A, one can also use an approach that is somewhat analogous to early studies in which resistance modulators were added to standard chemotherapy at the time of progression on chemotherapy [174-178]. Here, one would initiate treatment with A and would repeat a tumor biopsy once the tumor stopped shrinking with A or began to grow. Patients positive for X at that point might then be randomized to B alone vs continuing A and adding B to assess tumor regression and time to progression after initiation of B. One would continue A in one arm since A might have caused regression of a rapidly growing dominant clone, Y, permitting outgrowth of a more slowly growing sub clone X that is potentially sensitive to B, but if A is stopped, then the original Y clone might grow back rapidly and lead to progression on B alone. Such “flare” responses have been documented for patients who had responded to a targeted therapy and who had then had the targeted therapy stopped at tumor progression [179].

### Summary

While RCCTs have contributed to modest progress, they have also been inefficient, wasteful, and potentially misleading. We cannot sustain the huge costs (in dollars and in life-years lost) of our current approaches. We need to change clinical trial designs to reflect the fact that sensitizing mutations may be present in only a minority of patients with a given malignancy, we must put a very high premium on obtaining tissue from clinical trial patients, we should grant approval to single agents that achieve high rates of sustained response in defined groups of pretreated patients, and we must rethink how we approach trials of drug combinations. The primary endpoint of clinical trials needs to shift from the question “Is the therapy of benefit overall to the group” to the question “Who (if anyone) benefits?”

In summary, specific suggestions include the following:

1. A very high premium must be placed on tissue acquisition for patients participating in trials of new agents.

2. Do not do RCCTs in unselected patients unless the drug target is known to be present in most patients.

3. Do not use RCCTs in unselected patients to compare 2 agents with differing mechanisms of action.

4. For chemotherapy agents, we need to define the molecular characteristics of sensitive tumors.

5. Response is generally a more efficient endpoint than survival for discovering predictive biomarkers, and progression-free survival is also more efficient than overall survival.

6. To increase measurement precision, measure larger tumors or else measure multiple smaller lesions.

7. Available data suggest that in searching for predictive biomarkers, the proportion of patients who will be biomarker-positive will generally be approximately twice the size of the proportion responding or approximately 5-10% less than the proportion of patients achieving >10% reduction in tumor diameter.

8. Continuously variable (or graded) biomarkers are generally less helpful in guiding therapy choices than dichotomous biomarkers (present vs absent), unless there is a marked nonlinear relationship between biomarker expression and drug benefit (as with estrogen receptors in breast cancer).

9. If using continuously variable biomarkers that have a linear relationship with drug benefit:

a. Devise models that permit estimate of an individual degree of benefit for a given patient.

b. Do not use arbitrary cut points to divide patients into high-risk vs low-risk groups.

10. Archival tissue may be used for biomarkers in those without prior response to a targeted therapy.

11. A fresh biopsy should be done for biomarkers in patients with prior response to a targeted therapy.

12. While reliability of FFPE methodologies may be improving, carefully processed fresh biopsies may improve reliability of gene expression arrays and of some immunohistochemistry assessments.

13. Using biomarkers to select patients can markedly reduce required RCCT patient numbers and costs.

14. For uncommon malignancies with few driving mutations, current phase II methods may be appropriate.

15. For phase II trials in common malignancies with many potential driving mutations, either:

a. Select patients based on specific molecular characteristics, or

b. Markedly increase trial size to ensure capture of multiple subgroups, with suspension of accrual of individual subgroups if no activity is seen in the first few patients in that subgroup.

16. If using an overall survival endpoint, collect detailed information following discontinuation of study therapy, including response to subsequent interventions.

17. Approve drugs for marketing without RCCTs if single-agent treatment gives a high rate of sustained responses in heavily pretreated patients from a defined population.

18. In assessing new combinations:

a. Do not abandon a new drug based solely on a negative RCCT of a combination.

b. Use strategies that test whether the combination is actually better than sequential single agents.

c. Formulate a biologically based hypothesis as to what you expect to see with the combination.

d. Use a trial design that is appropriate to test your hypothesis.

e. If hypothesizing additivity or synergism:

i. Obtain baseline tissues to permit later identification of sensitive subgroups

ii. Randomize to A followed by B (A→ B) vs B followed by A (B → A) vs A+B

iii. Assess whether A+B is better than A → B and B → A with respect to:

1. Time to failure on last of the sequential drugs vs time to failure on A+B

2. Best response on either of the sequential drugs vs best response on A+B

f. If hypothesizing that B will prevent outgrowth of sub clone X that is resistant to A:

i. Obtain baseline tissue

ii. Randomize to A → B vs B → A vs A+B

iii. Assess total time to failure from start of therapy to last therapy on each arm

iv. Quantify X (and other potential resistance factors) in biopsies done at the time of tumor progression on A on any arm, or at a set time point after starting A on any arm.

g. If hypothesizing that B will be effective against sub clone X that is resistant to A:

i. Obtain baseline tissue and initiate treatment with A

ii. In patients with tumor regression on A:

1. Repeat biopsies when the tumor stopped shrinking or started growing on A

2. If positive for X, randomize to B alone vs continuing A and adding B

3. Assess response/further tumor regression with B

4. Assess time to progression after initiation of B.

## Abbreviations

EGFR: Epidermal growth factor receptor; FISH: Fluorescent in situ hybridization; FFPE: Formalin-fixed paraffin-embedded; HR: Hazard ratio; IHC: Immunohistochemistry; NSCLC: Non-small cell lung cancer; PFS: Progression-free survival; RCCT: Randomized controlled clinical trial; RECIST: Response criteria in solid tumors; TKI: Tyrosine kinase inhibitor.

## Competing interests

DJS: No competing interests related to current work. Unrelated to current work (last 36 months): Paid consultancy: Align2Action, Amgen, Transport Canada, SAIC, Trinity Partners LLC, Coleman Research Group, Frankel Group, Roche Canada; Grants paid to institution: AstraZeneca, Pfizer Canada, Roche Canada, NIH, US Department of Defense; Payment for lectures: University of Michigan/SWOG, Ventana, IASLC, University of Texas San Antonio, American Radium Society, AstraZeneca Taiwan, Pfizer Canada, 5^th^ International Pulmonary Congress; Royalties: Springer. RK: No competing interests related to current work. Unrelated to current work (last 36 months): Grants to self and to institution: Amgen, Aronex, AstraZeneca, Centocor, Concordia, Exelixis, GlaxoSmithKline, Hoffman LaRoche, Janssen, Merck, Novartis.

## Authors’ contributions

DJS was responsible for conception and design, acquisition of data, analysis and interpretation of data, drafting of the manuscript, and approval of the final manuscript. RK contributed to interpretation of data, manuscript revision, and approval of the final manuscript.

## Pre-publication history

The pre-publication history for this paper can be accessed here:

http://www.biomedcentral.com/1471-2407/13/193/prepub
